# Combined pulmonary thrombectomy and kidney embolization as bailout to thrombolysis: a case report

**DOI:** 10.1093/ehjcr/ytag200

**Published:** 2026-03-13

**Authors:** Jens Trøan, Martin Kirk Christensen, Mikkel Taudorf, Mikkel Bak, Mikkel Hougaard

**Affiliations:** Department of Cardiology, Odense University Hospital, J. B. Winsløws Vej 4, 5000 Odense C, Denmark; Department of Cardiology, Odense University Hospital, J. B. Winsløws Vej 4, 5000 Odense C, Denmark; Department of Radiology, Odense University Hospital, J. B. Winsløws Vej 4, 5000 Odense C, Denmark; Department of Radiology, Diagnostic Center, Copenhagen University Hospital - Rigshospitalet, Blegdamsvej 9, 2100 Copenhagen, Denmark; Dept of Clinical Medicine, University of Copenhagen, Blegdamsvej 3B, 2200 Copenhagen N, Denmark; Department of Anesthesiology and Intensive Care, Odense University Hospital, J. B. Winsløws Vej 4, 5000 Odense C, Denmark; Department of Cardiology, Odense University Hospital, J. B. Winsløws Vej 4, 5000 Odense C, Denmark

**Keywords:** Pulmonary embolism, Catheter-based thrombectomy, Embolization, Thrombolysis, High-risk pulmonary embolism, PERT, Case report

## Abstract

**Background:**

Systemic thrombolysis fails in around 8% of high-risk pulmonary embolism (PE) patients, and is associated with an increased risk of bleeding. Catheter-based aspirational thrombectomy (CAT) is emerging as a promising treatment option for PE, even when thrombolysis fails.

**Case summary:**

A 47-year-old woman, with short bowel syndrome and a history of previous pulmonary embolism presents with acute chest pain and haemodynamic instability. CT angiography showed central pulmonary embolism, and the patient was initially treated with acute thrombolysis. Despite this, the patient remained haemodynamically unstable and developed a life-threatening retroperitoneal bleed originating from the left kidney. A multidisciplinary team discussion was held among a PE cardiologist, interventional cardiologist, interventional radiologist, and an intensive care specialist. As the patient was considered too unstable for surgery, an emergency percutaneous embolization of the left kidney combined with CAT was planned. The renal arterial angiography showed diffuse ongoing capsular bleeding and was successfully embolized with particles (EmboSphere 300–500 um) and two microcoils in the main renal artery. Thereafter, bilateral thrombectomy was performed using the AlphaVac^®^ system, and several large thrombi were removed. The patient´s haemodynamic improved instantly, and she was discharged from the intensive care unit to the cardiac ward after 11 days.

**Discussion:**

In this case, an acute and life-threatening complication to systemic thrombolysis was successfully managed with acute combined percutaneous embolization and thrombus aspiration. This procedure required careful planning and cooperation involving multiple specialties, emphasizing the importance of multi-disciplinary team discussions in the management of complex PE patients.

Learning points

- Aspirational thrombectomy is an effective and safe treatment in complicated high-risk pulmonary embolism.
- Aspirational thrombectomy can be conducted in conjuncture with other invasive treatments, like kidney artery embolization, to improve efficacy and reduce risks.
- A coordinated effort between different specialties is important for optimal and time-effective treatment of pulmonary embolism, especially in patients with complications.

## Introduction

**Figure 1:**
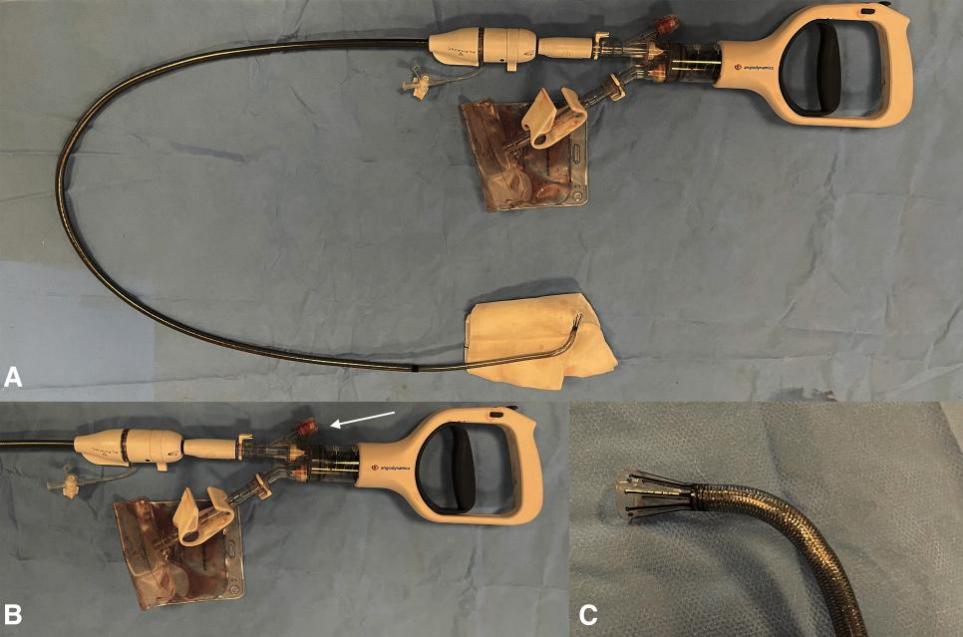
(*A*) Picture of the whole AlphaVac^®^ system. (*B*) Pictures of the AlphaVac^®^ handle with sideport (white arrows) and collection bags. (*C*) Picture of AlphaVac^®^ radiopaque nitinol funnel tip for aspiration of the thrombus. Created in BioRender. Trøan, J. (2026) https://BioRender.com/myuee29.

Pulmonary embolism (PE) is the third most frequent acute cardiovascular disease in Europe.^1^ Acute thrombolysis is the recommended treatment by ESC guidelines in haemodynamically unstable patients in the absence of contraindications. While thrombolysis is an effective treatment for high-risk PE, persistent haemodynamically instability and right ventricular (RV) dysfunction have been reported in up to 8% of high-risk patients.^2^ In addition, the risk of severe bleeding is as high as 9.9%.^3^ In patients with persistent PE after thrombolysis or with contraindications, percutaneous catheter-based therapy is a potential option, and currently holds a 2a recommendation in the ESC guidelines.^1^ Catheter-based therapy includes catheter-directed thrombolysis with or without mechanical disruption of the thrombus, or thrombectomy, either by mechanical, aspirational, rheolytic, or fragmentational techniques.^4^ The AlphaVac^®^ (Angiodynamics, Latham, NY, USA) is a catheter-based aspirational thrombectomy (CAT) device (*Figure 1*), which has shown promising results in the treatment of PE.^5^

In this case, we present a case of failed thrombolysis in a patient with pulmonary embolism complicated by a life-threatening kidney bleed that was successfully managed in a combined procedure with kidney artery embolization and coiling followed-by CAT.

## Summary figure

**Figure:**
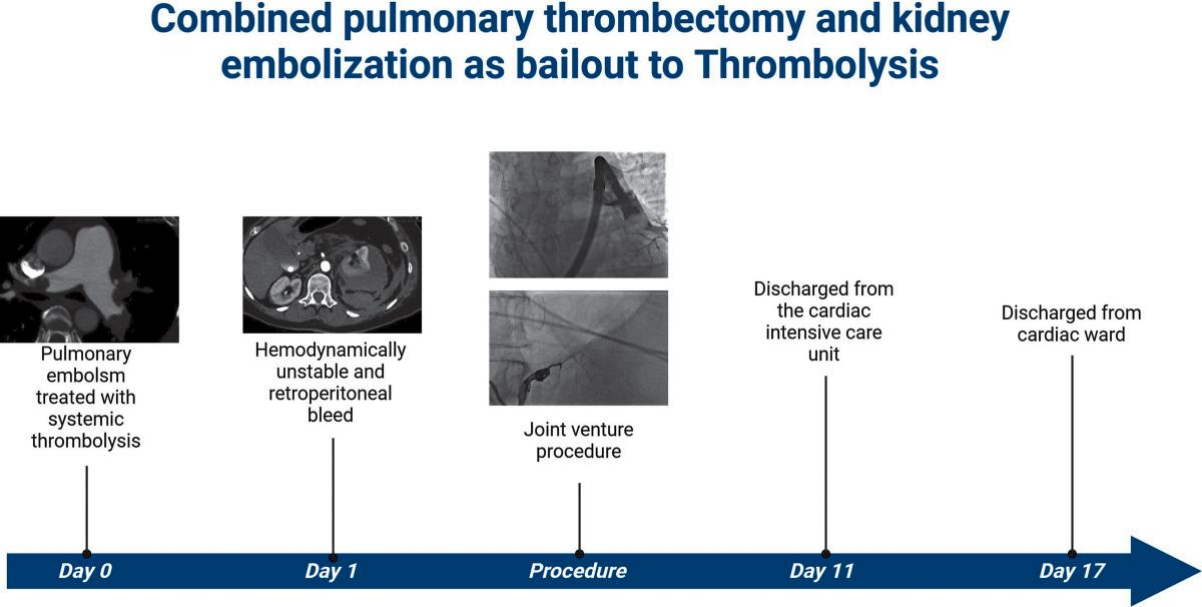


## Case presentation

**Figure 2:**
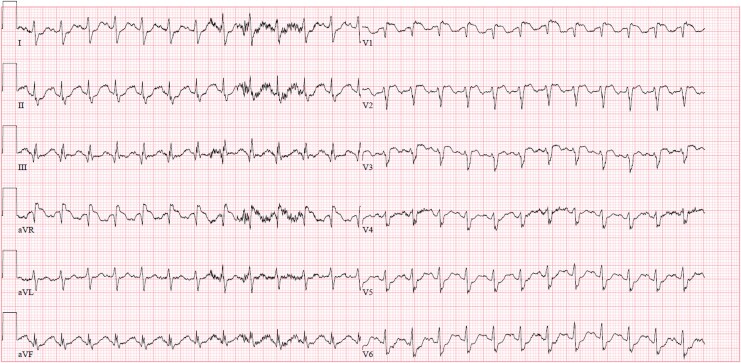
Electrocardiogram taken by the ambulance before hospital admission showing sinus tachycardia with ST-elevation in V1-V3 and in aVR.

**Figure 3:**
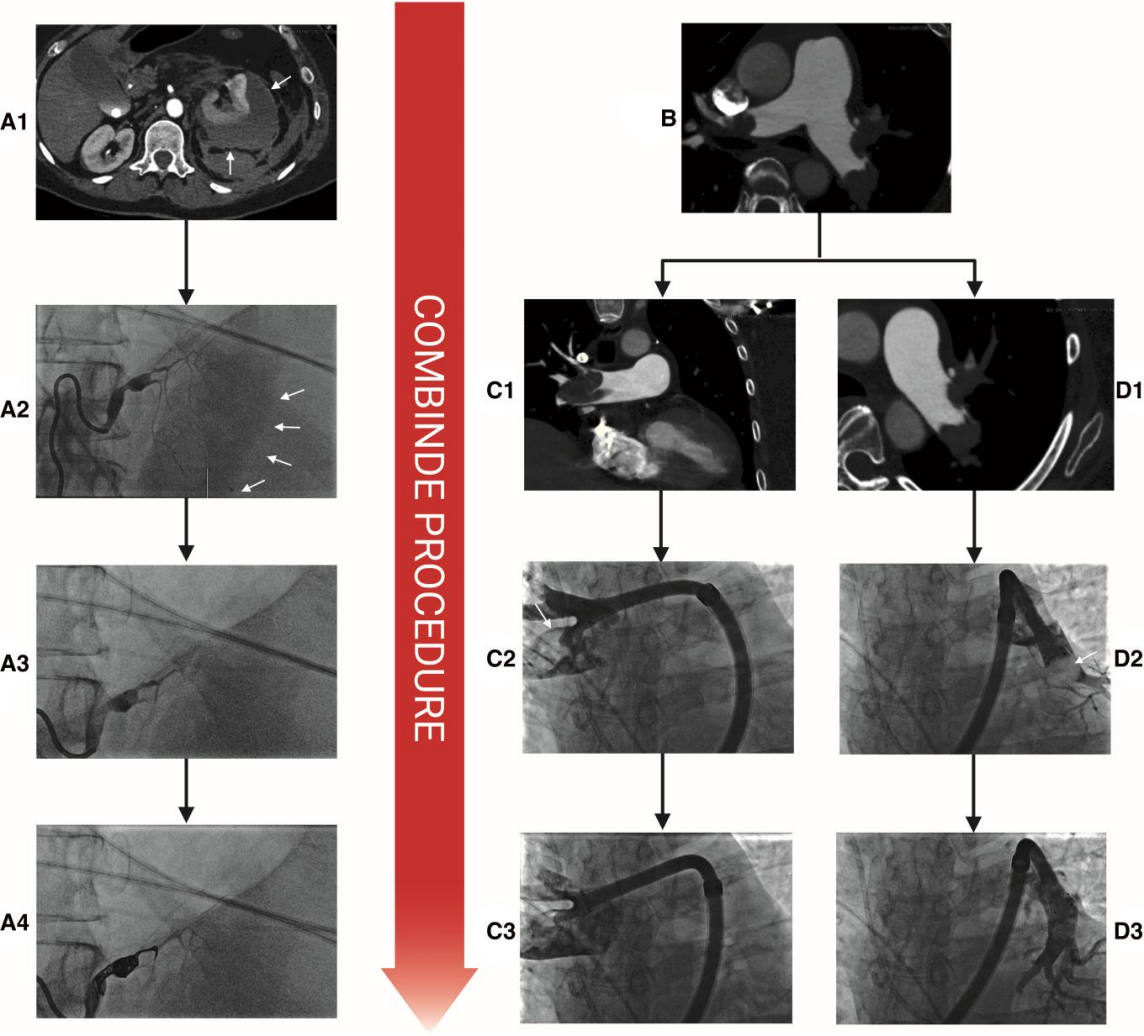
A1: CT scan of kidney haemorrhage (arrows pointing at haematoma), A2: renal angiography with diffuse microbleeding (white arrows), A3: renal angiography after injection of EmboSphere, A4: final renal angiography after embolization and coiling. B: CT scan showing bilateral central pulmonary embolism. C1: CT scan of the right pulmonary artery showing a large central thrombus, C2–3: pulmonary angiography showing thrombus (white arrow) before aspiration (C2) and after (C3) in the right pulmonary artery. D1: CT scan of the left pulmonary artery showing a large central thrombus, D2–3: pulmonary angiography showing thrombus (white arrow) before aspiration (D2) and after (D3) in the left pulmonary artery. Created in BioRender. Trøan, J. (2026) https://BioRender.com/ewu94n3.

A 47-year-old woman presented with chest pain and haemodynamic instability. She had a medical history of bipolar disorder, and five months prior to admission, underwent abdominal surgery for endometriosis complicated by bowel perforation necessitating reoperations that subsequently led to short bowel syndrome. During this period, the patient developed deep vein thrombosis and pulmonary embolism, which were treated with low-molecular-weight heparin injections; these were ongoing at the time of admission. The initial electrocardiogram showed sinus tachycardia with ST-elevations in V1-V3 and in aVR (*Figure 2*). Due to haemodynamically instability and suspicion of acute coronary syndrome, the patient was referred directly to our cath-lab for assessment. The patient presented with a blood pressure of 70/50 mmHg, an initial lactate of 5.9 mmol/L, requiring a dose of 0.2 µg/kg/min noradrenaline to maintain acceptable blood pressure. Bedside echocardiography showed acute RV failure with D-sign configuration and an increased tricuspid regurgitation gradient of 50 mmHg. The patient underwent emergent CT angiography, confirming central pulmonary embolism (*Figure 3*:B, C1 and D1). Due to the absence of any contraindications, haemodynamically instability, and limited availability of catheter-directed therapy, a ESC guideline-recommended management strategy of systemic thrombolysis was chosen for treatment of the high-risk pulmonary embolism. Thrombolysis was performed and the patient was transferred to the cardiac intensive care unit (CICU) for further monitoring. Initially, the patient responded to thrombolysis with a reduction in heart rate and lactate, but maintained a need for noradrenaline to maintain acceptable blood pressure.

**Figure 4:**
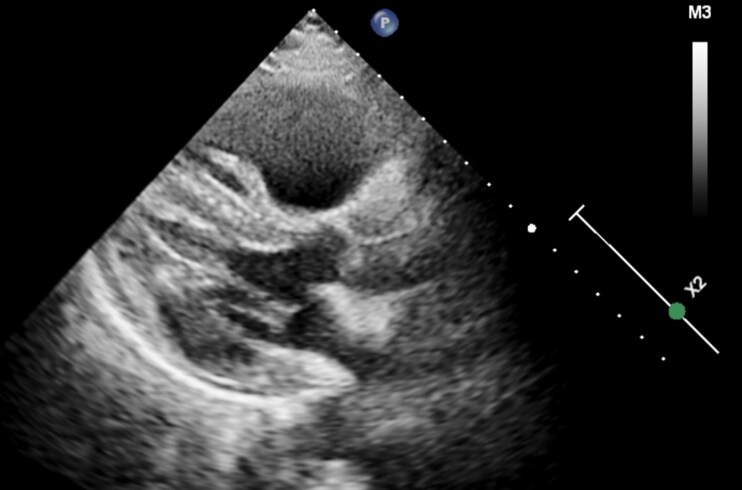
Echocardiography in the parasternal long-axis view, showing an enlarged right ventricle and an underfilled left ventricle.

During the second CICU day, the patient developed abdominal pain and an acute CT revealed a major retroperitoneal bleed originating from the left kidney with diffuse active contrast extravasation from the capsula of the kidney (*Figure 3*:A1). The patient deteriorated haemodynamically and required vasopressor treatment to maintain blood pressure and needed a total of four blood transfusions to make up for an acute reduction in haemoglobin from 8.1 mmol/L at admission to 4.6 mmol/L. The echocardiography showed persistent signs of RV failure in addition to an underfilled left ventricle (LV) (*Figure 4*). A multidisciplinary team discussion was conducted between a PE cardiologist, an interventional cardiologist, an interventional radiologist, and an intensive care specialist, who decided to perform a combined procedure of embolization of the left kidney as well as CAT. Due to the transfusion-requiring bleed to maintain an acceptable haemoglobin, the renal artery embolization was chosen as the initial procedure, with a plan to switch to CAT if the patient rapidly deteriorated.

**Figure 5:**
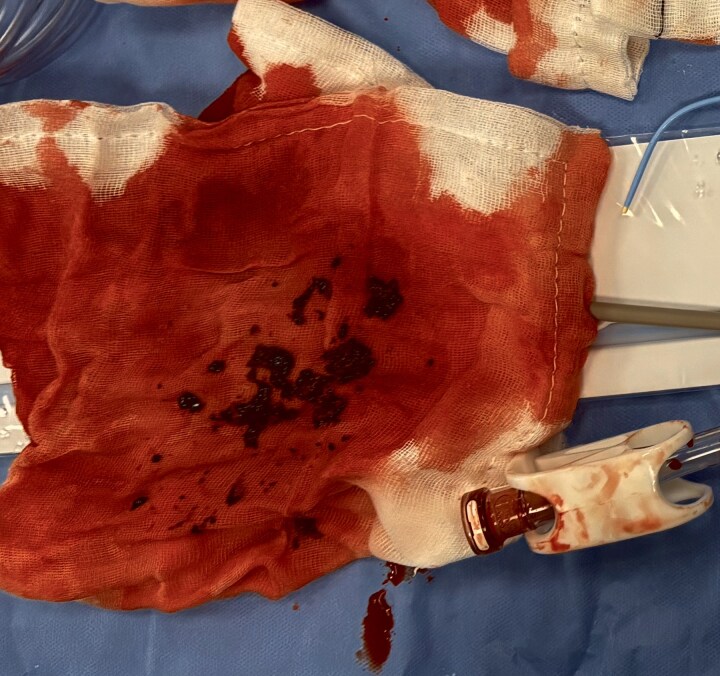
Picture from the procedure showing some of the aspirated thrombus.

The patient was intubated and sedated for the procedure, and was haemodynamically supported by infusions of noradrenaline, vasopressin, and adrenaline. The patient was anticoagulated by continuous heparin infusion of 1500 IE/h to obtain an activated partial thromboplastin time (APTT) of 45–50. Ultrasound-guided access of the left femoral artery was obtained, and a 5 Fr guiding catheter was placed in the left renal artery. Angiography showed microperforation with signs of ongoing bleeding (*Figure 3*:A2). A microcatheter was advanced and 3 mL of EmboSphere suspension (Merit Medical, South Jordan, UT, USA) was injected (*Figure 3*:A3). Afterwards, two Concerto microcoils (Medtronic, Minneapolis, MN, USA) of 6 mm/20 cm and 7 mm/30 cm were inserted and no signs of further bleeding were seen (*Figure 3*:A4). Thereafter, CAT was performed. Through a right femoral vein access, an angulated pigtail was advanced through the pulmonary valve before being advanced distally in the left pulmonary artery. After exchange to a stiff Amplatz guidewire, the 22 Fr AlphaVac^®^ sheath was advanced to the pulmonary truncus followed by the advancement of the AlphaVac^®^ system. The right pulmonary artery was engaged, showing large thrombi on the angiography, and several large thrombi were successfully aspirated (*Figure 3*: C2–3, *Figure 5*). The procedure was repeated on the left pulmonary artery, also with retrieval of thrombus material (*Figure 3*: D2–3). At the end of the procedure, the blood flow of the pulmonary artery was significantly improved.

Immediately following the procedure, the patient rapidly improved haemodynamically and could subsequently be reduced in vasopressors in the following days. Echocardiography showed improvement of RV function and normal filling of the LV. After a total of 11 days in the CICU the patient was transferred to the cardiac ward for further monitoring and treatment.

The patient’s condition improved during her hospital stay, and after 17 days she was transferred to the gastroenterological ward for further treatment of her Short Bowel syndrome and further optimization of her kidney disease. After a total of 55 days, the patient was discharged with normalized kidney function and was in her usual state of function at 3-month follow-up.

## Discussion

While systemic thrombolysis is an effective treatment in high-risk PE with hemodynamic instability, the optimal management when thrombolysis fails can be challenging. Catheter-based therapy is typically the preferred treatment strategy, with the availability of the different modalities differing greatly between centres. The AlphaVac^®^ is an 18 Fr large-bore vacuum-assisted CAT device comprising a distal funnel expanding to 33 Fr (*Figure 1*). There is still limited data regarding efficacy, with only one case report published on its successful use in treating PE.^6^ However, in the single-arm APEX-AV trial, the AlphaVac^®^ system showed a significant reduction in RV/LV ratio at 48 h post-procedure and a reduction in pulmonary artery pressure postprocedure.^5^ This case is, to the best of our knowledge, the first to report the use of the AlphaVac^®^ in the rescue treatment of failed thrombolysis high-risk PE.

Embolization and coiling are safe and effective treatments for ongoing bleeds. The procedure is also relatively fast and minimally invasive, minimizing both procedure time and the associated risks of larger surgery. In cases like ours, where a patient is haemodynamically unstable, a prolonged surgical procedure would be associated with a high risk of further complications, rendering embolization the only option to achieve haemostasis.

This case shows the feasibility of a combined catheter-based approach for management of haemostasis following failed thrombolysis and improvement in haemodynamics in acute life-threatening PE.

This case also emphasizes the importance of multi-disciplinary team discussions in PE patients, especially in cases with severe complications. The ESC guidelines encourage the development of a pulmonary embolism response team (PERT) including cardiologists, intensive care, radiologists, among others.^1^ In this case, the patient underwent life-saving intervention with both embolization and thrombus aspiration in the same procedure, which requires careful planning and cooperation between specialties that a PERT might facilitate.

## Conclusion

In patients with pulmonary embolism refractory to systemic thrombolysis and complicated by retroperitoneal bleeding, a combined catheter-based procedure with CAT and kidney arterial embolization and coiling was successfully conducted. A PERT team discussion is monumental in deciding the optimal treatment for patients with complicated PE.

## Data Availability

Data regarding this case is available upon reasonable request.

## Lead author biography

Dr Jens Trøan graduated from the University of Southern Denmark in 2023. He is a clinical researcher and medical doctor at the Department of Cardiology, Odense University Hospital. His areas of interest are catheter-based interventions, invasive coronary physiology, PCI, and clinical trials.

## Notes

**Consent:** Written consent, in line with the COPE guidelines, for the submission and publication of this case was obtained from the patient.
